# Age-related variation in thyroid function – a narrative review highlighting important implications for research and clinical practice

**DOI:** 10.1186/s13044-023-00149-5

**Published:** 2023-04-03

**Authors:** Peter N. Taylor, Andrew Lansdown, Justyna Witczak, Rahim Khan, Aled Rees, Colin M. Dayan, Onyebuchi Okosieme

**Affiliations:** 1 Thyroid Research Group Institute of Molecular and Experimental Medicine, C2 link corridor, UHW, Cardiff University School of Medicine, Heath Park, Cardiff, UK; 2 Department of Endocrinology, University Hospital of Wales, Cardiff, UK; 3 Neuroscience and Mental Health Research Institute, Cardiff University School of Medicine, Cardiff, UK; 4 Diabetes Department, Prince Charles Hospital, Cwm Taf Morgannwg University Health Board, Merthyr Tydfil, UK

**Keywords:** Thyroid function, TSH, FT4, Reference interval, Children, Adolescents, Aging, Elderly, T4, T3, Tri-iodothyronine, Thyroxine

## Abstract

**Background:**

Thyroid hormones are key determinants of health and well-being. Normal thyroid function is defined according to the standard 95% confidence interval of the disease-free population. Such standard laboratory reference intervals are widely applied in research and clinical practice, irrespective of age. However, thyroid hormones vary with age and current reference intervals may not be appropriate across all age groups. In this review, we summarize the recent literature on age-related variation in thyroid function and discuss important implications of such variation for research and clinical practice.

**Main text:**

There is now substantial evidence that normal thyroid status changes with age throughout the course of life. Thyroid stimulating hormone (TSH) concentrations are higher at the extremes of life and show a U-shaped longitudinal trend in iodine sufficient Caucasian populations. Free triiodothyronine (FT3) levels fall with age and appear to play a role in pubertal development, during which it shows a strong relationship with fat mass. Furthermore, the aging process exerts differential effects on the health consequences of thyroid hormone variations. Older individuals with declining thyroid function appear to have survival advantages compared to individuals with normal or high-normal thyroid function. In contrast younger or middle-aged individuals with low-normal thyroid function suffer an increased risk of adverse cardiovascular and metabolic outcomes while those with high-normal function have adverse bone outcomes including osteoporosis and fractures.

**Conclusion:**

Thyroid hormone reference intervals have differential effects across age groups. Current reference ranges could potentially lead to inappropriate treatment in older individuals but on the other hand could result in missed opportunities for risk factor modification in the younger and middle-aged groups. Further studies are now needed to determine the validity of age-appropriate reference intervals and to understand the impact of thyroid hormone variations in younger individuals.

## Introduction

Thyroid hormones play an important role in the development and maintenance of normal metabolic processes throughout life [1, 2]. Clinically, thyroid function is assessed by measuring thyroid stimulating hormone (TSH) and free thyroid hormone levels [3]. The complex inverse relationship between TSH and thyroid hormone levels renders TSH the more sensitive marker of overall thyroid status [4]. Euthyroidism or “normal” thyroid function is based on establishing the 95% confidence interval of TSH and thyroid hormone levels in individuals without thyroid disease. Whilst this statistical approach is a key component of conventional clinical practice, this “one size fits all” approach may need refining. It is well established in adults that there is narrower intra-individual variation in thyroid hormone parameters compared to the variation observed between individuals [5]. Furthermore, thyroid hormone levels are largely genetically determined [6–8] with similar genetic effects observed in children and adults [8]. Thus, the thyroid function of an individual may remain within the defined population range but fall outside their genetically determined set-point.

There is increasing evidence that thyroid status within the reference range is a risk factor for disease burden [9, 10] and that over 95% of rigorously screened individuals without thyroid disease or autoantibodies have TSH concentrations below 2.5 mIU/L [11]. These considerations have generated intense debate as to whether the existing approach to establishing thyroid reference ranges should be refined [12, 13]. Manipulation of reference-ranges is not a new concept. For example, the defined reference range for cholesterol is not related to its distribution in a population but instead to its 10-year cardiovascular mortality risk [14]. A similar approach for thyroid function is admittedly more complex as adverse risks exists both for high and low thyroid function whereas the key risk in cholesterol is seen with higher levels. Stratification of thyroid status reference ranges is nevertheless appealing.

**Fig. 1:**
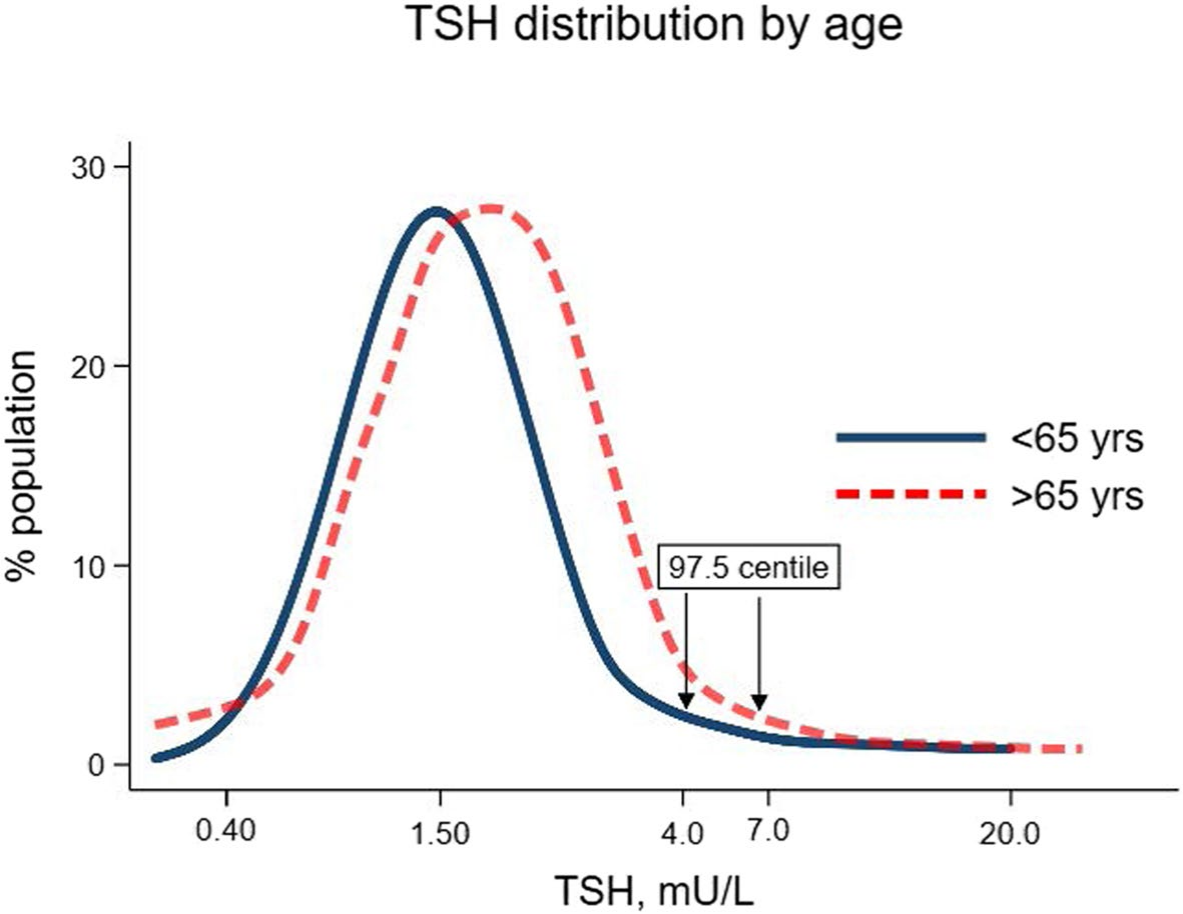
TSH distribution by age. “Legend: Figure is a schematic simple curve based approximately on data in studies which have shown an increase in TSH reference intervals with increasing age (reference 15–17). The X axis is not drawn to scale for illustrative purposes”

Compelling arguments can be made for age-specific, ethnicity specific, and pregnancy specific reference ranges [3]. Of these, age is particularly important as symptoms consistent with ageing such as tiredness and fatigue are potential but not strongly predictive features of hypothyroidism [3]. In addition, the normal TSH distribution curve is shifted to the right in the elderly (Fig. 1) and it is increasingly recognised that higher TSH levels may represent a normal part of ageing [15–17]. Thus, older individuals with marginal elevations of TSH may be inappropriately treated for hypothyroidism even though their thyroid function is within the normal range for their age-group. This may explain at least in part the lack of therapeutic benefit observed in older individuals with subclinical hypothyroidism [18]. There is also evidence that in individuals established on Levothyroxine, dose requirement falls with age [19, 20]. In contrast thyroid hormone levels may be higher in children and there is growing evidence that children and adolescents require higher Levothyroxine doses than adults [21].

**Fig. 2:**
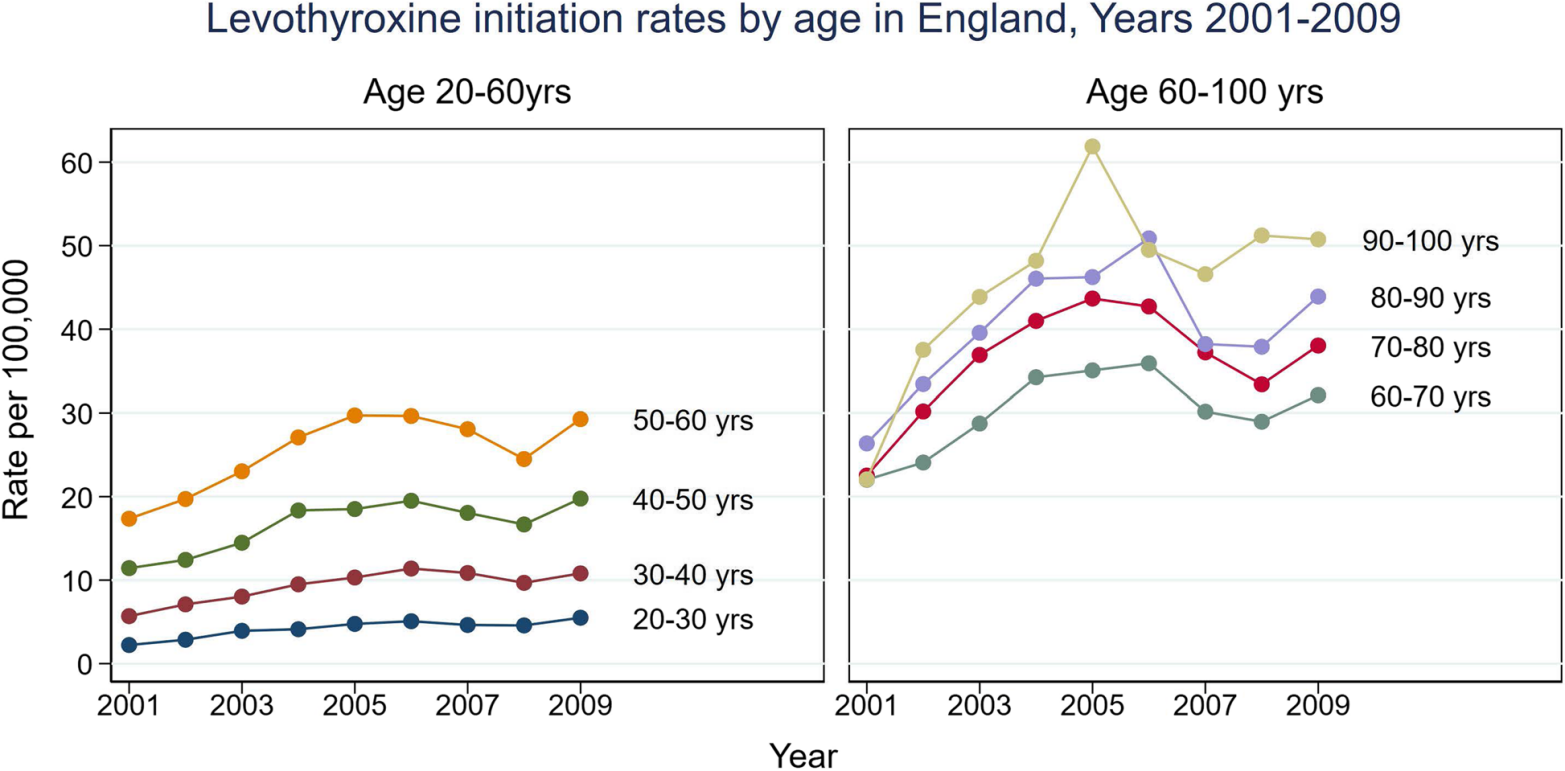
Levothyroxine initiation rates by age in England, Years 2001–2009. Legend: Figure adapted from data in reference 24

These considerations have implications for clinical practice as hypothyroidism is common globally and increases in prevalence with age [22, 23]. Furthermore, the TSH threshold for levothyroxine initiation has been falling in recent years with many older individuals increasingly started on Levothyroxine at borderline TSH levels [24]. In the UK, Levothyroxine initiation in the elderly has progressively increased with annual initiation rates ranging between 30 and 50 per 100,000 in individuals aged over 60 years (Fig. 2). Overall, the prevalence of hypothyroidism in the UK increased 50% from 2.3 to 3.5% between 2005 and 2014 [25]. Thus, the prevalence of hypothyroidism is set to increase given the ageing global population [26] together with widespread thyroid function testing [22]. A re-appraisal of the life-course of thyroid function is therefore timely and will have implications not only for clinical practice but also for clinical trials [27]. In this review we highlight the age-related changes in thyroid function that occur through childhood and adolescence as well as the variation in adulthood and older individuals and explore the implications of these changes for research and clinical practice.

## Methods

We undertook a narrative review of the literature and searched PubMed using a combination of the key words: thyroid function, TSH, FT4, FT3, TT4, TT3 reference range, reference interval, children, adolescents, aging, and elderly. We reviewed relevant articles published in the English language between January 2000 and February 2021. Our preference was for relevant original studies, including observational studies, clinical trials, and meta-analysis, that have shaped current knowledge and practice with respect to age-related variation in thyroid function. Changes to thyroid status and ideal population and trimester reference-ranges are beyond the scope of this paper and are dealt with by another excellent review of thyroid in pregnancy [28].

### Thyroid function in children

Thyroid hormones are responsible for normal childhood growth and development [1]. Uncorrected thyroid dysfunction is associated with growth disorders, delays in sexual maturation, and cognitive dysfunction in children [29]. In addition, variation in thyroid function within the normal population reference range in children is associated with negative effects on important outcomes such as blood pressure [30] lipid metabolism [31], and pubertal development [32]. Small studies in children indicate marked variation in thyroid hormone reference ranges such that adult reference intervals are not universally applicable to children [33–35]. A systematic review of reference range studies in children showed TSH upper limit ranges as wide as 2.36–6.45 mIU/L in 11–20-year-olds with free thyroxine (FT4) lower and upper limits of 7.0–14.2 and 15.5–31.5 pmol/L, respectively [36]. In addition, reference range variation was even more pronounced in the younger age brackets and grew narrower with increasing age. However, these variations may partly be due to differences in study parameters including age at sampling, assay methods, ethnicity, body mass index, iodine nutrition status, as well as the methodological approach for deriving the reference intervals.

#### TSH reference intervals in children

Cross-sectional studies generally show higher TSH upper limits in younger children (< 5 years) compared to older children, with a gradual decline in TSH concentration as the adult age is approached [33, 36–39]. In some studies, TSH concentration was higher in boys than in girls [38], was positively associated with body weight [40, 41], and showed ethnic-related variation. For example, TSH was higher in Caucasian children compared to children of African descent [36] or in Jewish versus non-Jewish children [41].

Two large longitudinal studies have analysed TSH profiles through adolescence in the same population [32, 42] (Table 1). In the first of these studies, we reported a rise in TSH levels from age 7 to 15 years in 884 participants of the Avon Longitudinal Study of Parents and Children (ALSPAC) [32]. In addition, we found a higher TSH level in boys than in girls but with no difference in the slope of increase between the sexes. The second study was a recent longitudinal analysis of participants in the Brisbane Longitudinal Twin study in which thyroid function was measured at age 12, 14, and 16 years [42]. The study reported no change in TSH concentration from age 12 to 14 years but with a subsequent increase from 14 to 16 years. Although different age ranges were examined in these studies, these two longitudinal findings are largely consistent in the increase in TSH across the pubertal years. Applying the adult reference ranges to children in both studies would have misclassified TSH as either abnormal or normal in 3-6% of the population [32, 42].

**Table 1:** Longitudinal studies of thyroid function in children and adolescents

Author and year	Cohort, number	Ages sampled	Assay Used	TSH change	FT4 change	FT3 change
Taylor, 2017 [32]	ALSPACUK, *n* = 884	7, 15 yrs	Cobas e601 (Roche)	*7 to 15 yrs* Increased by 0.12 mIU/L. No sex difference in change.	*7 to 15 yrs* Decreased by 0.16 pmol/L. No sex difference in change.	*7 to 15 yrs* Decreased by 0.48 pmol/L. Greater decline in girls than boys.
Campbell, 2020 [42]	BLTS Australia, *n* = 1499	12, 14, 16 yrs	Abbott ARCHITECT	*12 to 14 yrs* Boys: no change. Girls: no change.	*12 to 14 yrs* Boys: no change. Girls: increased by 0.30 pmol/L.	*12 to 14 yrs* Boys: Increased by 0.29 pmol/L Girls: Increased by 0.07 pmol/L
				*14 to 16 yrs* Boys: increased by 7.2%. Girls: increased by 4.9%.	*14 to 16 yrs* Boys: increased by 0.64 pmol/L. Girls: increased by 0.42 pmol/L.	*14 to 16 yrs* Boys: decreased by 0.62 pmol/L. Girls: decreased by 0.53 pmol/L.

*Legend*: Longitudinal changes were derived from linear mixed models adjusted for age, sex, puberty, and body mass index (Taylor [32]), and for age, puberty, and body mass index (Campbell [42]); *ALSPAC* Avon Longitudinal Study of Parents and Children, *BLTS* Brisbane Longitudinal Twin Study

#### FT4 and FT3 reference intervals in children

Previous cross-sectional studies have indicated that free triiodothyronine (FT_3_) falls and FT_4_ rises from age 4 years [43–45]. In a population-based Danish cohort of 2411 healthy 6–19-year-old children, median values of FT4 and FT3 fell with increasing age for both sexes with the decline most pronounced around the time of puberty, age 11-15 years [46]. In Indian children of a similar age group FT3 and FT4 also showed an inverse association with age, although this relationship was only significant in girls [47, 48].

In our cohort study in which thyroid function was measured at ages 7 and 15 years in the same children, we identified substantial changes in thyroid hormones, particularly FT3 [32] (Table 1). At age 7 children had a higher FT3 than children at age 15, and 23% of children at age 7 had FT3 above the adult reference-range. Girls had a higher baseline FT3 than boys and a greater slope of decline. For FT4, these changes were less pronounced. FT4 at age 7 years was slightly higher than at 15 years and was higher in girls compared to boys. In both hormones there was greater variability at age 7 compared to age 15 years with convergence towards the adult reference ranges with age [32]. In the study by Campbell et al, FT3 in girls showed minimal change from 12 to 14 years and then declined sharply from 14 to 16 years whereas in boys it increased from age 12 to 14 and decreased by age 16 years [42]. FT4 on the other hand increased from age 14 to 16 years in both sexes. Application of the adult FT3 reference range would have misclassified 35% of 12-year-old girls and 58% of 14-year-old boys as high [42].

FT3 in particular may have important influences on childhood development especially at puberty. In our data, higher FT_3_ levels at age 7 in boys and girls were associated with attainment of a more advanced pubertal stage at age 13, but no clear effect was seen with TSH and FT4 [32]. Likewise, the study by Marwaha and colleagues in Indian children showed that the onset of puberty was accompanied by a rise in FT3 together with a corresponding fall in FT4 [44]. Intriguingly, a Mendelian Randomization study in our cohort identified that higher levels of fat mass appeared to result in higher levels of FT3 particularly in younger children [49]. This effect was less apparent in older children and may be diminished in adults. However, a similar effect is still observed in obese adults who often have higher FT3 levels and a higher FT3:FT4 ratio than the general population [50]. It is likely that the observed relationship between FT3 and BMI is driven by fat mass, however the mechanism for this is unclear. It is possible that increased fat mass induces a response from the HPT axis to preferentially increase FT3 levels or that increased brown adipose tissue which is rich in DIO2 increases conversion of FT4 to FT3. More research is needed in this area.

Overall, these findings suggest that variation in thyroid function in children is more plastic than previously envisaged. Further studies are needed to clarify the pituitary-thyroid axis in childhood and adolescence. The variations observed for FT3 may be particularly relevant to children with congenital hypothyroidism. It is uncertain whether treated hypothyroid children have adequate FT3 levels for optimal timing of puberty and other developmental processes. It is noteworthy that hypothyroidism diagnosed in the immediate pre-pubertal years can cause delays in puberty [51]. It is also possible that relative lack of FT3 may contribute to the cognitive deficits observed in children with congenital hypothyroidism despite adequate Levothyroxine replacement [52]. Manipulating FT3 levels in children with congenital hypothyroidism to mirror the levels seen in children without hypothyroidism would be conceptually attractive but challenging to safely implement. Unlike Levothyroxine which has extensive safety data the long-term safety of Liothyronine has not been established in children [53, 54]. However, the development of longer acting T3 preparations might offer a safer means of replicating normal thyroid status without the potential risks of short-acting Liothyronine on cardiovascular and bone health.

### Thyroid function in the general adult population

Most laboratories report a TSH interval between 0.4 and 4.5 mIU/L in the disease-free adult population. This broad and somewhat arbitrary reference interval represents the gold standard measure of thyroid dysfunction in adults. In reality the positively skewed TSH distribution means that most individuals have a TSH between 0.4 and 3.0 mIU/L (Fig. 1) [15]. In older individuals on the other hand, the TSH distribution curve shifts to the right such that the upper TSH reference limit is as high as 7.0 mIU/L. Thus, lowering the TSH upper limit would classify 15–20% of the elderly population as subclinical hypothyroidism, a condition for which treatment benefits are unproven [55]. A meta-analysis of randomised controlled trials (RCTs) showed no symptomatic benefits of Levothyroxine in adults with subclinical hypothyroidism [56]. However, the meta-analysis included a significant proportion of elderly participants (mean age > 65 years) with mild TSH elevations which may reflect the aging process rather than intrinsic thyroid disease [18, 57]. Furthermore, some of the RCTs were conducted in asymptomatic individuals with mild TSH elevations (3.5–10 mIU/L). Given that Levothyroxine is increasingly offered to individuals with subclinical hypothyroidism [24], it is not surprising that symptomatic patients are under-represented in clinical trials since they would already have been started on treatment.

#### Implications of thyroid hormone variation within the normal reference range

Studies in euthyroid populations offer an alternative approach for investigating the effects of thyroid function variation in adults [10]. Unlike studies in subclinical thyroid disease, the lack of treatment in euthyroid cohorts allows convenient analysis of large datasets without the confounding effects of treatment. A 2013 meta-analysis of observational studies revealed that even within the population reference range, variations in thyroid hormone levels were associated with a range of adverse cardiovascular, metabolic, and bone outcomes [10]. In essence, high-normal TSH, was associated with adverse cardiovascular and metabolic outcomes whereas low-normal TSH was associated with adverse bone outcomes. In particular higher levels of TSH were associated with worsening of both blood pressure [58–60] and lipid levels [61, 62] although these effects were modest. In addition, higher TSH levels within the reference-range were associated with higher all cause [63] and cardiovascular mortality [63, 64]. Associations with blood pressure and lipid levels were also present in children [30, 31] highlighting the influence of TSH on cardiovascular risk factors throughout life.

Changes in body weight have also been linked with thyroid function variation within the population reference-range. Higher TSH levels were associated with increased BMI [65, 66], and in longitudinal studies, individuals with rising TSH levels over time exhibited greater weight gain [67, 68]. In contrast FT4 was strongly negatively associated with BMI [65]. In addition, lower thyroid function within the population reference range was associated with an increased risk of non-alcoholic fatty liver disease in middle aged individuals [69]. This effect persisted despite adjusting for age, sex, use of lipid modifying drugs and other cardiovascular risk factors [69]. Similar cross-sectional analyses have identified that lower TSH levels and higher levels of FT4 within the population reference-range are associated with increased risk of developing osteoporosis [70–73] and suffering subsequent fractures [71, 74] in post-menopausal women.

However, it is important to note that these associations are based on observational cross-sectional data and therefore prone to the limitations of unmeasured or residual confounding as well as the possibility of reverse causation. Also, these studies were undertaken in samples with predominantly middle-aged participants and so will not be applicable to older individuals for whom a differential impact of thyroid function on health outcomes has been observed in the euthyroid population.

Mendelian Randomization studies which are more resistant to confounding and reverse confounding [75] where the life time effect of an exposure (e.g. thyroid function) on an outcome (e.g. cardiovascular disease) is evaluated using genetic variants associated with the exposure as the instruments. The genetic architecture of thyroid function is increasingly understood [7, 8] making Mendelian Randomization an attractive research method here. Mendelian Randomization has shown how modest variation in thyroid status is associated with stroke and coronary artery disease [76]. Mendelian Randomization studies also can show mechanistic insight, a recent study demonstrated that the well-known association between variation in thyroid function and atrial fibrillation is for an important part mediated by height [77].

### Thyroid function in older individuals

The global population is aging at unprecedented rates driven by increasing life expectancy [26]. By the year 2050, it is estimated that 16% of the world population will be aged 65 years and over [26]. The prevalence of thyroid dysfunction increases with age and older individuals with thyroid dysfunction suffer significant morbidity and quality of life impairment [22]. Hyperthyroidism in the elderly increases the risk of osteoporosis [78], cardiac arrhythmias, and heart failure [78, 79], while hypothyroidism predisposes to coronary artery disease, lipid disorders, and neurocognitive impairment [3]. In addition, both hyperthyroidism and hypothyroidism increase frailty in the elderly and are associated with the development of myopathy [80], gait disorders [81], and fractures [10, 82].

#### Thyroid function variation with age

Evidence from animal and human studies show that thyroid function declines with age [23]. The hypothalamic pituitary thyroid axis is altered with age and older individuals have been shown to exhibit a blunted TSH response to thyroid hormone deficiency [4, 23]. It has also been postulated that TSH bioactivity may be reduced with aging or that the thyroid may be less sensitive to TSH [83]. Studies in rats show reduced thyroid hormone secretion and reduced tissue expression of thyroid hormones in the liver and kidney [84]. In addition, hepatic expression of the deiodinase enzyme, DIO1, and the mono-carboxylate transporter (MCT-8) are reduced while DIO3 expression is increased [85, 86]. These changes appear to be tissue specific as pituitary expression of DIO1, DIO2, and triiodothyronine (T3) are increased while muscle and brain DIO3 expression remains unchanged or reduced [84].

#### Cross-sectional studies

**Fig. 3:**
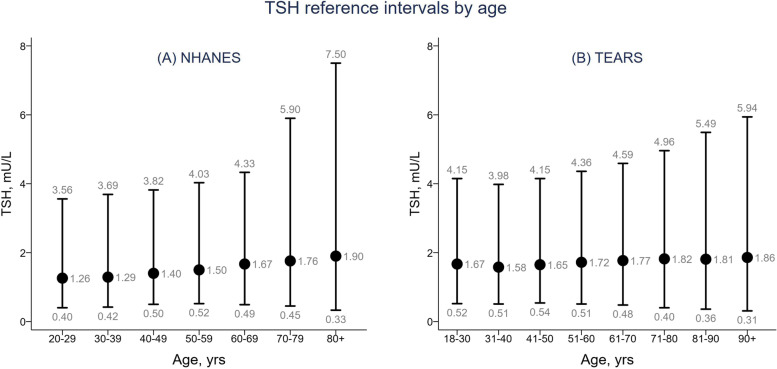
TSH reference intervals by age. Change in TSH reference ranges with age based on data from The National Health and Nutrition Examination Survey (NHANES) (**a**, reference 15) and the Thyroid Epidemiology, Audit and Research Study (TEARS) (**b**, reference 17)

Several large cross-sectional studies in iodine-replete thyroid disease-free populations have reported higher TSH concentrations in older individuals (Fig. 3), [15, 17, 87, 88]. The National Health and Nutrition Examination Survey (NHANES) III reported an age-related increase in TSH with the 97.5 centile exceeding 7.0 mIU/L in individuals aged over 80 years [15, 87]. These studies also show that the reference range becomes wider as age increases, with lower 2.5 centiles in the older age groups (Fig. 3). However, not all studies have confirmed these changes. Studies from Holland [89], India [90], Germany [91], China [92] as well as older data from Italy [93] and the UK Whickham community survey failed to show TSH elevations with age. However, variation in antibody status and inclusion of individuals with TPO antibody positivity, will influence results and ranges In these studies.

The reasons for these discrepancies are unclear but may be related to differences in cohort selection, analytical factors, iodine nutrition, and the genetic characteristics of participants. The TSH assays used in older surveys were less sensitive than current assays making direct comparisons unreliable. Environmental iodine status influences the TSH reference intervals with higher 97.5 centiles in areas with normal or excess iodine nutrition compared to iodine deficient areas [92]. Studies in populations with iodine deficiency have failed to show an increase in TSH [91, 93–95]. A study in elderly Dutch men showed no age-related changes in TSH and FT4 but reported lower FT3 levels together with an increase in reverse T3 (rT3) with age [89]. Regarding genetic factors, up to 67% of the circulating TSH and thyroid hormone concentrations are genetically determined [6] and several candidate genes have been implicated including the genes for phosphodiesterase 8B, DIO1, and the TSH receptor [96].

#### Longitudinal studies

A number of longitudinal studies have provided useful information on the natural history of thyroid function with aging (Table 2) [97–101]. Two of these studies showed an increase in TSH levels with age. The first, a study of 908 individuals in the Busselton health survey, showed that TSH levels increased after a 13-year period but with no significant change in FT4 [97]. The second study was an analysis of thyroid function in the Cardiovascular Health Study All-Stars Study which showed a TSH increase of 13%, FT4 increase of 1.7%, and a 13% fall in total T3 concentrations over a 13-year period [98]. Three other longitudinal studies in contrast did not confirm age-related TSH increases [99–101]. A longitudinal analysis of 9402 participants from the Rotterdam study showed no change in TSH but an increase was observed for FT4 concentrations after 6.5 years of follow up [99]. In a 5-year follow up study of 2936 participants from the UK Birmingham Elderly Thyroid Study, TSH was shown to be stable over time with 96% of euthyroid individuals (TSH < 4.5 mIU/L) remaining euthyroid at follow up [101].

**Table 2:** Longitudinal studies of thyroid function in adults and elderly

Author and year	Cohort and country	Number	Mean age, years	Follow up years	Assay Used	Key findings
Bremner, 2012 [97]	Busselton health survey, Australia	908	46	13	Immulite 2000 chemiluminescent analyzer (Siemens)	Mean TSH increased from 1.49 to 1.81 mIU/L; No change in FT4
Waring, 2012 [98]	Cardiovascular Health Study All-Stars Study, USA	843	85	13	Elecsys 2010 analyzer (Roche)	Mean TSH increased from 2.60 to 2.94 mIU/L; FT4 increased from 1.20 to 1.22 pmol/l; Total T3 decreased from 116.9 to 102.0 pmol/l
Chaker, 2016 [99]	The Rotterdam study, Netherlands	1225	67	6·5	electrochemiluminescence immunoassay, “ECLIA” (Roche)	No change in TSH; FT4 increased by a mean of 4.5 pmol/L
Mammen, 2017 [100]	Baltimore Longitudinal Study of Aging, USA	640	66	7	immunoassay Vista Chemiluminescence,(Siemens)	No change in means of TSH, FT4, and FT3. Changes in TSH more common in older participants
Roberts, 2018 [101]	Birmingham Elderly Thyroid Study, UK	2936	77	5	electrochemiluminescence immunoassay, “ECLIA” (Roche)	No change in TSH or FT4.

In the third study, Mammen and colleagues followed up 640 participants over an average period of 7 years in the Baltimore Longitudinal Study of Aging [100]. Although mean TSH was higher in older individuals at baseline, the TSH remained stable in the majority of participants. Time-related changes were more common in older individuals, associated with decreased survival and showed a heterogeneous pattern of change that included progression to primary thyroid disease as well as non-pathological TSH elevations [100]. While the differences in these studies are difficult to reconcile, it is worth noting that negative studies had shorter follow up periods (5-7 years) compared to positive studies (13 years). Taken together, these data suggests that thyroid status may decline in some older individuals, at least in iodine-replete Caucasian populations. However, the consistency of this decline across older populations will require further study.

#### Implications of thyroid function variation in the elderly

There is evidence that age-related changes in thyroid function serve as an adaptive mechanism with beneficial long-term consequences. Animal studies show negative correlations between thyroid hormone levels and longevity, with the longest survival observed in mice with lower thyroid hormone levels [102]. Epidemiological studies confirm survival advantages, both for low thyroid hormones and high TSH levels within the normal reference range [103, 104]. Studies in the oldest centenarians indicate that individuals with genetically higher TSH concentrations have enhanced longevity [105]. In addition, older adults with low-normal FT4 exhibit better mobility and fitness levels compared to those with normal FT4 [106]. A large individual-patient data meta-analysis also identified that high TSH levels within the normal population range was associated with a lower risk of stroke [107]. In addition, elderly persons with the lowest TSH tertile at baseline had more concurrent depressive symptoms and were at two-fold increased risk of subsequent depression than those with the highest tertile [108].

It is unclear whether the apparent survival benefit associated with lower thyroid status is due to a change in thyroid status in the elderly, survivor bias, or an indication of mild thyroid failure due to longevity or late auto-immunity as the presence of TPO antibody positivity increases with age [22]. This distinction is important because a change in thyroid status has clear implications for acceptable thyroid function and treatment targets in the elderly. In the absence of age-stratified reference intervals, individuals with age-related changes in TSH could be misdiagnosed as thyroid dysfunction and exposed to potentially harmful treatments. Analysis of the NHANES data estimated that 70% of older patients with TSH above the laboratory reference interval had values within their age-specific reference brackets [16]. However, it is worth noting that some studies have shown a more nuanced pattern of change with progression to thyroid failure and non-pathological TSH elevations in equal proportions [100]. TPO antibody positivity is unlikely to tbe the sole explanation for higher TSH levels in the elderly as the NHANES study identified that only 40% of the elderly with a TSH above 4.5 mU/L were TPO antibody positive [16] A study from Western Australia suggested that age-specific TSH reference intervals exerted only a negligible impact on the diagnosis of thyroid dysfunction in the elderly population [109]. In this study, only about 1–2% of individuals aged over 65 years were misclassified using the general adult reference intervals [109].

Thus, the need for age-specific reference intervals should be population-specific and should be based on data generated locally or extrapolated from a compatible population in terms of iodine nutrition and ethnicity. Whether age-specific treatment targets are required for individuals on thyroid hormone replacement also merits consideration. If the TSH increase in the elderly is indeed beneficial, then it is plausible that older individuals are over-treated by aiming for lower reference range TSH values. Also, Levothyroxine affects the T3:T4 ratio and individuals on levothyroxine have relatively low FT3: FT4 ratios [110]. Studies assessing the effects of age on the T3:T4 ratio, and its consequences on well-being would be particularly welcome given the large numbers of individuals on thyroid hormone replacement. Genetic association studies using variation in *DIO1* which is associated with FT3:FT4 ratio but not TSH [111, 112] may also provide insights.

It is also important to briefly mention macroTSH, which may cause of 1% of cases of SCH. It is a rare condition caused by binding of TSH to plasma proteins, usually immunoglobulins which results in falsely elevated TSH measurements but normal T3 and T4 levels and may lead to inappropriate LT4 treatment [113]. Whilst rare, macroTSH is thought to be more common in the elderly and therefore will have a greater relative effect in this group [114].

Lastly, there are implications for the design of future trials in subclinical hypothyroidism. Such trials will need to consider age-appropriate definitions of thyroid dysfunction so as to exclude individuals with non-pathological TSH elevation. The differential effect of age on TSH reference intervals may to some extent explain the discrepancies seen in the outcomes of existing trials. A 2008 meta-analysis by Razvi et al showed that subclinical hypothyroidism was associated with increased risk of cardiovascular mortality in studies conducted in younger (< 65 years) populations but not in studies in older individuals (> 65 years) [115]. Likewise, trials of levothyroxine therapy in elderly individuals with mild subclinical hypothyroidism have not shown clinical benefits [18, 57] and it is plausible that TSH elevations in these individuals did not represent intrinsic thyroid failure.

### Consideration of immunoassay interference and clinical implications

It is worth highlighting that whilst immunoassay platforms are currently the method of choice for the measurement of thyroid function tests [116], there are limitations; as well as macro-TSH, interference from biotin, thyroid hormone autoantibodies, heterophilic antibodies, antistreptavidin antibodies, anti-ruthenium antibodies, and heterophilic antibodies can occur [116]. The prevalence of some of these conditions has been reported to approach 1% and given the frequency of thyroid function testing [117] this may impact numerus individuals. Care needs to be taken therefore if results are inconsistent with a patient’s clinical picture.

## Conclusion

In this review, we have highlighted considerable age-related variation in thyroid function reference intervals over the course of life. In iodine sufficient populations, longitudinal changes in TSH are U-shaped, with higher TSH levels observed at the extremes of life. FT3 on the other hand falls with age and appears to play a role in pubertal development. Furthermore, there is a differential effect of aging on the health consequences of thyroid hormone variations. Declining thyroid function in the elderly may confer survival advantages whereas younger individuals with low-normal thyroid status have an increased risk of adverse atherogenic and cardiovascular outcomes. These findings support the need for age-appropriate thyroid hormone reference intervals. However, further studies will be needed to confirm specific patterns of change in different populations. In addition, trials of subclinical hypothyroidism are needed in younger individuals as the observations in older individuals cannot be extrapolated to the younger population.

Lastly, the current evaluation of thyroid function based on circulating TSH and thyroid hormones are imperfect, and more specific biomarkers of tissue hypothyroidism [118] are needed. There is evidence that TSH normalisation does not necessarily optimise tissue thyroid status in individuals on levothyroxine [119]. A meta-analysis of 65 studies that compared 1878 levothyroxine-treated patients to 14,493 healthy controls revealed that both total-cholesterol and LDL-cholesterol remain elevated, after serum TSH has been normalized [120] likely reflecting lower hepatic T3 status. Given the pleiotropic effect of thyroid hormones on metabolism and gene expression, metabolomics and transcriptomics [121] are emerging as attractive research targets for studying the tissue action of thyroid hormones.

## Data Availability

No data has been made available as it is a narrative review.
