# Correction to: Age-related variation in thyroid function – a narrative review highlighting important implications for research and clinical practice

**DOI:** 10.1186/s13044-023-00163-7

**Published:** 2023-05-30

**Authors:** Peter N. Taylor, Andrew Lansdown, Justyna Witczak, Rahim Khan, Aled Rees, Colin M. Dayan, Onyebuchi Okosieme

**Affiliations:** 1 Thyroid Research Group Institute of Molecular and Experimental Medicine, UHW, Cardiff University School of Medicine, C2 link corridor, Heath Park, Cardiff, UK; 2 Department of Endocrinology, University Hospital of Wales, Cardiff, UK; 3 Neuroscience and Mental Health Research Institute, Cardiff University School of Medicine, Cardiff, UK; 4 Diabetes Department, Prince Charles Hospital, Cwm Taf Morgannwg University Health Board, Merthyr Tydfil, UK

**Correction to:** ***Thyroid Res*** **16, 7 (2023).**

10.1186/s13044-023-00149-5

Following publication of the original article [1], the authors identified an error in the Competing interests section.

The incorrect Competing interests section is:

**Competing interests**.

The authors declare no competing interests.

The correct Competing interests section is:

**Competing interests**.

Peter Taylor declares that he is Associate Editor of *Thyroid Research* and that the article was assigned to another Editor to assume responsibility for overseeing peer review. This submission was subject to the exact same review process as any other manuscript submitted to the journal.

The Competing interests section has been updated above and the original article [1] has been corrected.
